# Impact of the 2018 revised Pregnancy Prevention Programme by the European Medicines Agency on the use of oral retinoids in females of childbearing age in Denmark, Italy, Netherlands, and Spain: an interrupted time series analysis

**DOI:** 10.3389/fphar.2023.1207976

**Published:** 2023-08-17

**Authors:** Carlos E. Durán, Judit Riera-Arnau, Shahab Abtahi, Romin Pajouheshnia, Vjola Hoxhaj, Magdalena Gamba, Ema Alsina, Mar Martin-Perez, Patricia Garcia-Poza, Ana Llorente-Garcia, Diana Gonzalez-Bermejo, Luisa Ibánez, Mònica Sabaté, Xavier Vidal, Elena Ballarín, Gabriel Sanfélix-Gimeno, Clara Rodríguez-Bernal, Salvador Peiró, Aníbal García-Sempere, Francisco Sanchez-Saez, Valentina Ientile, Ylenia Ingrasciotta, Claudio Guarneri, Matilde Tanaglia, Michele Tari, Ron Herings, Eline Houben, Karin Swart-Polinder, Emily Holthuis, Consuelo Huerta, Rosa Gini, Giuseppe Roberto, Claudia Bartolini, Olga Paoletti, Giorgio Limoncella, Anna Girardi, Giulia Hyeraci, Morten Andersen, Sarah Brøgger Kristiansen, Christine Erikstrup Hallgreen, Olaf Klungel, Miriam Sturkenboom

**Affiliations:** ^1^ Julius Center for Health Sciences and Primary Care, Department of Data Science and Biostatistics, University Medical Center Utrecht, Utrecht, Netherlands; ^2^ Department of Clinical Pharmacology, Vall Hebron Institut de Recerca (VHIR), Universitat Autònoma de Barcelona (UAB), Barcelona, Spain; ^3^ Division of Pharmacoepidemiology and Clinical Pharmacology, Faculty of Science, Utrecht Institute for Pharmaceutical Sciences, Universiteit Utrecht, Utrecht, Netherlands; ^4^ Agencia Española de Medicamentos y Productos Sanitarios, Madrid, Spain; ^5^ The Foundation for the Promotion of Health and Biomedical Research of Valencia Region, Health Services Research Unit (FISABIO - HSRU), Valencia, Spain; ^6^ Department of Biomedical and Dental Sciences and Morpho-functional Imaging, University of Messina, Messina, Italy; ^7^ Section of Pharmacology, Department of Diagnostics and Public Health, University of Verona, Verona, Italy; ^8^ Caserta Local Health Unit, Caserta, Italy; ^9^ PHARMO Institute, Utrecht, Netherlands; ^10^ Department of Public Health and Maternal and Child Health, Faculty of Medicine, Universidad Complutense de Madrid, Madrid, Spain; ^11^ Agenzia Regionale di Sanità Della Toscana (ARS), Florence, Italy; ^12^ Pharmacovigilance Research Center, Department of Drug Design and Pharmacology, Faculty of Health and Medical Sciences, University of Copenhagen, Copenhagen, Denmark; ^13^ Copenhagen Centre for Regulatory Science, Department of Pharmacy, Faculty of Health and Medical Sciences, University of Copenhagen, Copenhagen, Denmark

**Keywords:** oral retinoids, isotretinoin, contraceptive measures, dermatologic conditions, acne, risk minimisation measures, pregnancy prevention programme

## Abstract

**Background:** In March 2018, the European pregnancy prevention programme for oral retinoids was updated as part of risk minimisation measures (RMM), emphasising their contraindication in pregnant women.

**Objective:** To measure the impact of the 2018 revision of the RMMs in Europe by assessing the utilisation patterns of isotretinoin, alitretinoin and acitretin, contraceptive measures, pregnancy testing, discontinuation, and pregnancy occurrence concomitantly with a retinoid prescription.

**Methods:** An interrupted time series (ITS) analysis to compare level and trend changes after the risk minimisation measures implementation was conducted on a cohort of females of childbearing age (12–55 years of age) from January 2010 to December 2020, derived from six electronic health data sources in four countries: Denmark, Netherlands, Spain, and Italy. Monthly utilisation figures (incidence rates [IR], prevalence rates [PR] and proportions) of oral retinoids were calculated, as well as discontinuation rates, contraception coverage, pregnancy testing, and rates of exposed pregnancies to oral retinoids, before and after the 2018 RMMs.

**Results:** From 10,714,182 females of child-bearing age, 88,992 used an oral retinoid at any point during the study period (mean age 18.9–22.2 years old). We found non-significant level and trend changes in incidence or prevalence of retinoid use in females of child-bearing age after the 2018 RMMs. The reason of discontinuation was unknown in >95% of cases. Contraception use showed a significant increase trend in Spain; for other databases this information was limited. Pregnancy testing was hardly recorded thus was not possible to model ITS analyses. After the 2018 RMM, rates of pregnancy occurrence during retinoid use, and start of a retinoid during a pregnancy varied from 0.0 to 0.4, and from 0.2 to 0.8, respectively.

**Conclusion:** This study shows a limited impact of the 2018 RMMs on oral retinoids utilisation patterns among females of child-bearing age in four European countries. Pregnancies still occur during retinoid use, and oral retinoids are still prescribed to pregnant women. Contraception and pregnancy testing information was limited in most databases. Regulators, policymakers, prescribers, and researchers must rethink implementation strategies to avoid any pregnancy becoming temporarily related to retinoid use.

## Introduction

Retinoids are vitamin A derivatives that regulate cell differentiation, proliferation, and apoptosis. All retinoids are considered highly teratogenic and must not be used during pregnancy. Retinoic acid embryopathy includes central nervous system abnormalities (hydrocephalus, microcephaly), external ear abnormalities (anotia, microtia, or absent external auditory meatus), cardiovascular abnormalities (septal wall and aortic defects), facial dysmorphia (cleft palate), eye abnormalities (microphthalmia), and thymus gland and bone abnormalities (Soprano and Soprano, 1995). In pregnancies ending in birth, congenital malformations after the exposure to oral retinoids have been reported to be as high as 28%. (Dai et al., 1992).

Acitretin, alitretinoin, and isotretinoin are the oral retinoids authorised in the European Union (EU). To minimise risk, risk minimisation measures (RMMs) have been requested by regulators and pregnancy prevention programmes (PPPs) put in place worldwide (Shin et al., 2011; Panchaud et al., 2012; Pinheiro et al., 2013). In 2003, a PPP was implemented in the EU for oral retinoids. The effectiveness of this PPP has been reviewed and despite a reduction in number, pregnant women exposed to retinoids have continued to occur, raising concerns about compliance and effectiveness of PPPs in clinical practice (Teichert et al., 2010; Crijns et al., 2012; Zomerdijk et al., 2014; Raguideau et al., 2015; Henry et al., 2016). In June 2018, the Pharmacovigilance Risk Assessment Committee (PRAC) required harmonisation of warnings about teratogenic risks (Referral under Article 31 of Directive 2001/83/EC) and asked for a post-authorisation safety study to assess the effectiveness of revised additional RMMs in females of child-bearing age. According to the 2018 measures by PRAC (European Medicines Agency, 2018) (Box 1), oral retinoids are contraindicated in females of child-bearing potential for indications such as acne, eczema and psoriasis, unless the conditions of the PPP are fulfilled (Retinoids, 2018). The main changes between the prior and updated PPP consist of updated dissemination materials (a patient´s card, a prescriber and pharmacist checklist) and updated Summary of Product Characteristics, including the risks of neuropsychiatric disorders. The updated PPP stresses key aspects such as the requirement for women of childbearing potential to be clearly informed about the teratogenic risk of oral retinoids, the need for monthly or trimestral pregnancy testing, and the use of at least one (preferably two) complementary forms of contraception while taking retinoids (European Medicines Agency, 2018). The implementation of specific elements of the 2018 PPP were agreed at national level.

BOX 1 **Pregnancy prevention programme and implementation dates of RMM by country.**

**Summary of pregnancy prevention programme by PRAC 2018:**

• Individual patient and prescriber discussion should take place to guarantee patient engagement, discuss therapeutic options, and ensure the patient understanding of the risks and the measures needed to minimise the risks.
• The hazards and necessary precautions associated with retinoid use during pregnancy are presented in the risk acknowledgement form and the patient reminder card, which should be provided to the patients.
• Pregnancy testing should be performed prior to initiation of treatment, ideally monthly during treatment and after stopping treatment.
• The patient should be capable of complying with an effective contraceptive treatment/method, without interruption during the entire duration of treatment with oral retinoids acitretin, alitretinoin, isotretinoin and for 1 month [3 years for acitretin] after the end of treatment.
  ○ At least one effective method of contraception (preferably a user independent form such as an intra-uterine device or implant) or two complementary forms of contraception including a barrier method should be used.
• The prescribers must follow the Prescribers’ checklist (which includes the previous points) and ensure that the patient has understood and acknowledged the risks of congenital malformations, including the magnitude of these risks for children exposed to a retinoid *in utero*.
• The pharmacists should follow the Pharmacists’ checklist and ensure women understand the risk of being exposed to an oral retinoid means and comply with regular follow-up visits (with maximum 30-day supply prescriptions) and regular pregnancy testing.
• In case of pregnancy while using the oral retinoids acitretin, alitretinoin, or isotretinoin, the treatment must be stopped, and the patient must be immediately referred to a physician specialised or experienced in teratology for evaluation and advice.

**Implementation dates of 2018 RMM in countries included in this study:**

  • Denmark: 16/07/2018 to 11/10/2018
  • Italy: 08/08/2018 to 02/10/2018
  • Netherlands: 10/08/2018 to 12/12/2018
  • Spain: 24/07/2018 to 01/12/2018

The aim of this study was to measure the impact of the 2018 revision of the RMMs in Europe by assessing healthcare professionals’ compliance through the utilisation patterns of oral retinoids (isotretinoin, alitretinoin and acitretin) and the reasons of retinoid´s discontinuation, contraceptive measures, pregnancy testing, and pregnancy occurrence concomitantly with retinoid prescription, before and after the PPP revision was implemented.

## Patients and methods

### Study design, data sources and setting

**FIGURE 1:**
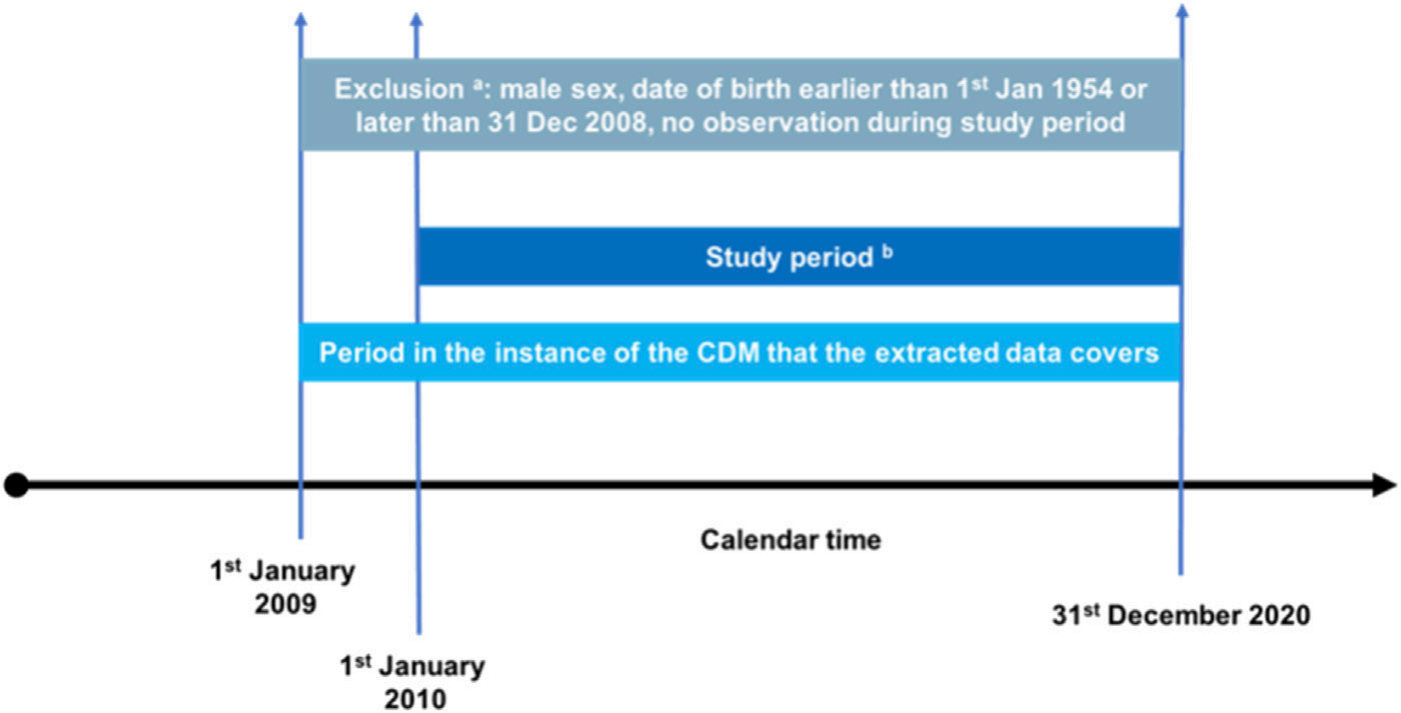
Query diagram to illustrate treatment episode construction and treatment discontinuation definition of oral retinoid and contraceptive exposures. **(a)** Subjects may be excluded during initial data processing if they meet the general exclusion criteria. This will help to reduce data volume. DAPs may apply these exclusion criteria during their ETL, but they must report this (it will also be detected when assessing at patient flow). Subjects with any record of male sex will be excluded. **(b)** Time series of cross-sectional analyses is performed over this period (monthly analyses). The final month will begin December 2020.

An interrupted time series analysis study was conducted to test the impact of the 2018 RMMs (box 1). Women of childbearing potential (12–55 years of age) from 01 January 2010 to 31 December 2020 were included. Figure 1 shows the population selection diagram. Data from six electronic healthcare databases (EHDs) from 4 European countries were used: i) the Italian Tuscany Regional Health Agency (IT-ARS), it consists of a regional administrative database including information on outpatient and community pharmacy dispensing records, and hospital and emergency room admissions, linked to a birth registry; ii) the Italian Caserta Local Health Agency Record Linkage Database (IT-Caserta), which is an administrative database with data from outpatient pharmacy dispenings, emergency and hospital discharge diagnoses, and also ambulatory information (data from IT-Caserta varied widely from 2010–2014 and could be utilised from 2015 onwards only); iii) the Spanish *Base de datos para la Investigación Farmacoepidemiológica en Atención Primaria* (ES-BIFAP), with information from the primary healthcare setting (GP visits, diagnostic and laboratory procedures, prescriptions and dispensations, and specialist referrals) (Maciá-Martínez et al., 2020); iv) the Spanish Valencian health system Integrated Database (ES-VID) through FISABIO, including information from primary and specialist ambulatory care, the emergency department, hospital admissions, prescription and dispensing of medicinal products, and a birth registry (García-Sempere et al., 2020); v) the Dutch PHARMO Database Network (NL-PHARMO), including GP and hospital diagnosis, prescriptions from GP setting only and a perinatal registry (Kuiper et al., 2020); and vi) in Denmark, University of Copenhagen provided access to an existing dataset of the Danish Nationwide registers including the Danish National Prescription Registry (DK-DNR) from 2010 to 2018, which includes administrative and clinical registers. We could not obtain pregnancy and hospital data due to delays in data delivery after the coronavirus disease 2019 (COVID-19) outbreak. For further details, see Supplementary Table S1. The study protocol is publicly available in the EU PAS register (EUPAS31095).

### Exposure

The main exposures of interest were the oral retinoid containing medicines, i.e., isotretinoin for acne (ATC code D10BA01), acitretin for psoriasis (ATC code D05BB02), and alitretinoin for eczema (ATC code D11AH04). Additionally, exposure to contraceptive measures was retrieved for assessment of appropriate contraception coverage.

Treatment episodes of retinoids and contraceptives were defined as the treatment periods from initiation to discontinuation of therapy, within the whole study period (see Supplementary Figure S1). Treatment initiation was defined as the record date of the first prescription or dispensing. When both prescription and dispensing dates were available, dispensing date was prioritised. A permissible gap of 30 days between episodes was implemented; for instance, if there was a gap of less than 30 days between the end of one episode and the next, the episodes were combined, and the person was assumed to be exposed during that gap. Treatment discontinuation was defined as not receiving any other prescription or dispensing of the same drug within 90 days after the last prescription/dispensing.

### Outcomes

The main outcomes of interest to measure the impact of the 2018 RMMs were i) patterns of use (prevalence, incidence), discontinuation rate and reason for discontinuation of oral retinoids; ii) contraception use prior to start of and during retinoid use; iii) pregnancy testing prior to the first retinoid prescription or dispensing and during retinoid use; iv) switching to alternative medicinal products (not reported in this manuscript) and v) the occurrence of pregnancy during retinoid use and initiation of a retinoid during the course of a pregnancy.

*Reasons for discontinuation* were classified as *pregnancy wish* if a female was prescribed folic acid within the 90 days following discontinuation of alitretinoin or isotretinoin; as *pregnancy* if a pregnancy event was observed within the 90 days following discontinuation of the retinoid; as *adverse drug reaction* (ADR) if a diagnosis for a pre-defined retinoid-associated ADR was recorded within the 90 days following discontinuation of the retinoid. To map for depression as a potential ADR, prescriptions/dispensings of antidepressants were used as proxy. See Supplementary Table S2 for ADRs associated to retinoids. The default reason for discontinuation was set as unknown (discontinuation without a recorded ADR, folic acid prescription/dispensing or a recoded start of pregnancy).

*Contraception use* was defined as the evidence of at least one user-independent method (permanent or non-permanent), or a hormone-based method combined with a barrier method, although barrier methods cannot be assessed reliably from EHDs since these are over-the-counter products. Contraception monthly rates were calculated, as well as episodes of contraceptive coverage. We assessed the proportion of retinoid treatments starting within a contraception treatment episode, and also contraception starting within 90 days prior to the start date of an oral retinoid *.*

*Pregnancy testing* was defined as any record or healthcare professional-witness of a pregnancy test. Recording of pregnancy testing was assessed within 90 days prior and 90 days after a retinoid treatment initiation. *Pregnancy* was properly identified across databases. We applied a pregnancy algorithm developed by Gini et al. (2022), which builds directly on top of a published algorithm for detecting pregnancies in databases (Matcho et al., 2018). Briefly explained, the proposed pregnancy algorithm allows the identification of pregnancies from 4 streams of information: perinatal or birth registries, administrative data banks using diagnosis codes, European registry of congenital abnormalities (EUROCAT) and a tailored-combined stream, which uses additional data from medical diagnoses. The algorithm first identifies pregnancies from any possible records and subsequently establishes the start and end date of pregnancy by processing all the available information on a hierarchical manner. Hierarchy is based on how identification of start and end dates of the pregnancy was performed. Four colour categories are used: a colour code green was assigned when both pregnancy start date and pregnancy end date were recorded in the database; yellow shows that pregnancy end date was recorded but pregnancy start date was imputed; blue indicates that pregnancy start date was recorded in the database and pregnancy end date was imputed; and the colour code red represents a pregnancy event for which both pregnancy start and end dates were imputed. Hence, green shows the highest certainty and red indicates the lowest one to ascertain a pregnancy start and end date from databases. In the main analysis, we used only pregnancies identified with the colour code green or yellow. More information about the applied pregnancy algorithm is publicly available at https://github.com/ARS-toscana/ConcePTIONAlgorithmPregnancies. For the identified pregnancies, first prescriptions/dispensings of oral retinoids during a unique pregnancy event were detected. Pregnancies exposed to retinoids were validated by each data access provider (DAP).

### Statistical analyses

Source and study population from each database were described in numbers and person-time by age, and calendar year. Monthly incidence rates (IR) of retinoid use were estimated as the number of new users per month, divided by the number of women at risk such month (person-months). We considered an incident use when no prescription or dispensing was present during a lookback-period of 1 year prior to the cohort entry month; if there had been a treatment exposure that overlapped with the current month, it was classified as prevalent use. Monthly prevalence rates (PR) were estimated and defined as the number of female retinoid users of childbearing age during a calendar month, divided by the total number of females of childbearing age of such calendar month. To assess IR discontinuation, the frequency of discontinuation divided by number of retinoid users from the previous quarter was calculated. IR and PR were stratified by type of retinoid/indication.

Pregnancy testing was analysed by means of incidence of witnessed pregnancy testing by person-month in women of childbearing age, and the monthly proportion of retinoid episodes with a pregnancy test within 90 days prior and within 90 days after the start of the retinoid. Contraception was studied by calculating the monthly proportion of retinoid prescription/dispensing with a recorded contraception method 90 days before starting the oral retinoid, and through the monthly proportion of retinoid prescription/dispensing that occurred within an established period of contraception coverage.

The occurrence of a pregnancy event related to a retinoid exposure has been assessed in two ways: first, we counted the monthly occurrence of the start of a pregnancy during a retinoid treatment episode; second, we counted the monthly occurrence of a retinoid prescription/dispensing during a pregnancy time window. Since all oral retinoids have a teratogenic risk period despite being discontinued (i.e., 1 month for alitretinoin and isotretinoin, and 3 years for acitretin) but follow-up time was short after the implementation of the RMMs in 2018, we added 1 month to the actual retinoid treatment episode end (also for acitretin). Counts were aggregated for the pre-intervention period and the post-intervention period and pregnancy rates were calculated per 1,000 retinoid users.

Sensitivity analyses were conducted i) anticipating the study end date to February 2020 to exclude the study period affected by the COVID-19 pandemic, which is known to have impacted healthcare seeking behaviour and collection of prescriptions and dispensings, and ii) redefining the discontinuation gap from 90 to 30 days and iii) adding red pregnancies (imputation of pregnancy start and end date) to the analysis in IT-ARS.

### Interrupted time series (ITS) analysis

Segmented generalised least squares regression analysis was used to compare monthly pre-intervention (2010–2018) and post-intervention (2018–2020) estimates in each of the tested outcomes: i) incident and prevalent retinoid use, ii) incidence of pregnancy testing compliant prescriptions, iii) incidence of contraception use, and iv) treatment discontinuation. The model included changes in level and trend after the implementation of the RMMs. The period following the promulgation of the RMMs to its actual implementation was excluded according to the dates indicated by each country, box 1. Seasonal patterns were adjusted in the model. Key assumptions of generalised least squares regression models were tested. Autocorrelation was tested with the Durbin-Watson statistic (Durbin and Watson, 1950) and graphically with autocorrelation function plots. Autoregressive structures were modelled to account for autocorrelation. Regression coefficients and *p*-values were estimated.

### Data management and quality assessment

This study was conducted in a distributed manner using the ConcePTION Common Data Model (CDM) and common analytics. This CDM has been described in full elsewhere (Thurin et al., 2022). The generic ConcePTION CDM is able to handle rich and non-standardised data vocabularies. A series of steps need to be taken. The DAP converts the study specific data into the ConcePTION CDM and runs quality checks on completeness (level 1), consistency (level 2), and distribution and trend of study variables over time (level 3). With this procedure we were able to assess fitness-for-purpose of such data, to assess its linkage, and to benchmark with existing literature to evaluate its external validity. The quality check scripts are publicly available on https://github.com/UMC-Utrecht-RWE. A study specific R-script was centrally developed to transform the study variables. The script was sent to all data sources to be run locally. DAPs transferred the aggregated results to YODA platform, a secure research repository at Utrecht University.

## Results

A total of 10,714,182 women of childbearing age (12–55 years of age) from the six participating DAPs were included in the study population. Table 1 describes the baseline characteristics of the study population and the retinoid exposed subpopulation by data source. The overall median follow-up time ranged from 7.6 years in DK-DNR (data available only until 2018) to 11.0 years in ES-VID. A total of 88,992 women of childbearing age used an oral retinoid at any point during the study period. Mean age at start of follow-up ranged between 18.9 [IQR 8.5 years] in IT-Caserta to 22.2 [IQR 11.1 years] in IT-ARS. Most retinoid users were in the younger age bands. The oral retinoid most frequently used was isotretinoin.

**TABLE 1:** Baseline characteristics (at start of follow-up) of the study population and persons using oral retinoid during follow-up in the different databases in Denmark, Italy, Netherlands, and Spain.

Country, and total active population in data source			Denmark, national registries^a^		Italy, ARS -Tuscany		Italy, Caserta		Netherlands, PHARMO	Spain, BIFAP		Spain, VID Valencia	
Size of the population (females 12–55 years of age), N			1,575,216		1,117,251		319,962		591,500	5,066,393		2,043,860	
Median follow-up, years (IQR)			7.6 (3.9)		8.2 (6.8)		9.9 (5.1)		10.0 (2.4)	9.4 (6.4)		11 (6)	
Mean age at start of follow-up date, years (SD)			30.8 (13.0)		33.8 (13.3)		31.5 (13.5)		29.8 (13.3)	31.7 (13)		31.4 (13.2)	
Study population, number (%) by age category	12- <21 years		447,233 (30.0)		244,952 (22.9)		88,126 (27.5)		188,176 (31.8)	1,212,120 (23.9%)		531,492 (26.0)	
	21- <31 years		307,839 (20.7)		181,458 (16.2)		60,946 (19.0)		121,343 (20.5)	1,107,176 (21.9%)		419,315 (20.5)	
	31-<41 years		308,224 (20.7)		279,584 (25.0)		71,955 (22.5)		121,455 (20.5)	1,272,723 (25.1%)		498,517 (24.5)	
	41–55 years		426,611 (28.6)		411,257 (36.8)		98,935 (31.0)		160,526 (27.1)	1,474,374 (29.0%)		594,536 (29.0)	
Female oral retinoid users, 12–55 years of age, N			40,834		6,930		5,565		4,225	15,981		15,457	
Mean age at start of follow-up, years (SD)			21.4 (9)		22.2 (11.1)		18.9 (8.5)		21.4 (10.6)	21.4 (10.6)		20.3 (10.2)	
Oral retinoid users, number (%) by age category at start of follow_up	12- <21 years		24,552 (60.1)		4,105 (59.2)		3,971 (71.4)		2,535 (60.0)	9519 (59.6)		10,071 (65.2)	
	21- <31 years		9,407 (23.0)		1,358 (19.6)		1,023 (18.4)		859 (20.3)	3436 (21.5)		2,866 (18.5)	
	31-<41 years		4,810 (11.8)		715 (10.3)		337 (6.1)		479 (11.3)	1,659 (10.4)		1,399 (9.1)	
	41–55 years		2065 (5.0)		752 (10.9)		234 (4.2)		352 (8.3)	1,367 (9.0)		1,121 (7.3)	

^a^ Baseline numbers in Denmark are calculated from a different source (please see Supplementary Table S1).

### Use and discontinuation patterns of oral retinoids

In general, IRs of oral retinoid use in females of childbearing age were below 1 per 1,000 person-months (PM). Overall IR of new users of any of the three retinoids over the study period were 0.22/1000 PM in DK-DNR, 0.06/1000 PM in NL-PHARMO, 0.16/1000 PM in IT-Caserta, 0.06/1000 PM in IT-ARS, 0.07/1000 PM in ES-VID, and 0.03 new users per /1000 PM in ES-BIFAP. Monthly IR and PR showed that retinoid prescriptions/dispensings have a strong seasonal pattern, with decreasing rates in the summer months, especially in southern European countries.

**FIGURE 2:**
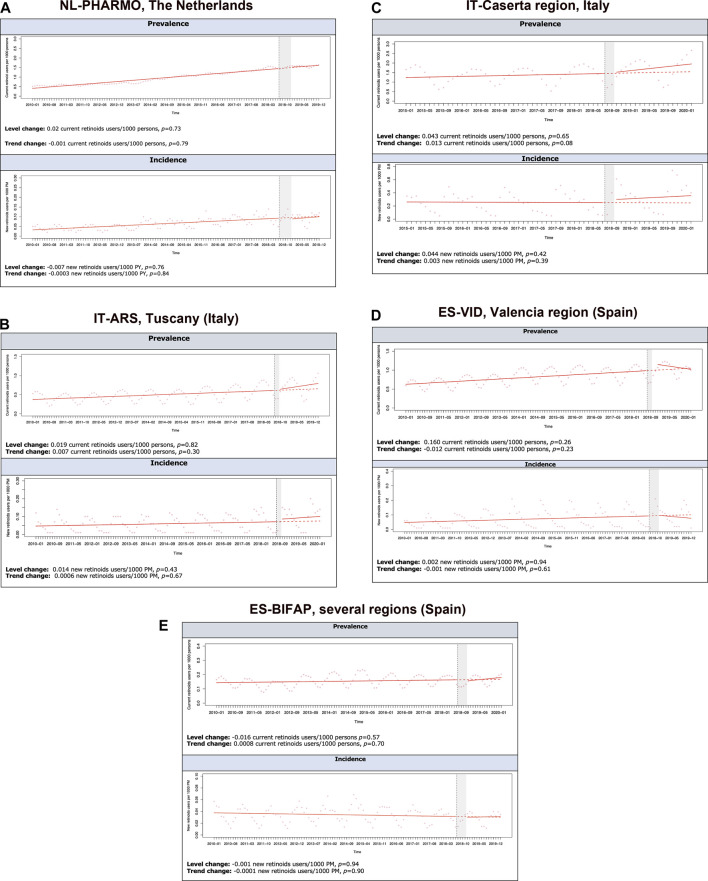
Interrupted time-series analyses (ITSA) on prevalence (current users/1,000 persons) and incidence (new users/1,000 person-months rates of retinoid use in female subjects of childbearing potential per database between 2010–2020, excluding COVID-19 pandemic period. **(A)** NL-PHARMO, Netherlands. **(B)** IT-ARS,Tuscany (Italy). **(C)** IT-Caserta region, Italy. **(D)** ES-VID, Valencia region (Spain). **(E)** ES-BIFAP, several regions (Spain).

Figures 2A–E show the results of the ITS analyses on IR and PR of oral retinoids, by censoring the COVID-19 pandemic period. For all data sources, prescription/dispensing retinoids’ monthly PR increased slightly over time, except in ES-BIFAP. We found no statistically significant change in incidence or prevalence of retinoid use, neither immediately (level) nor over time (trend) in women of childbearing age after implementing the 2018 RMMs. We could not model the ITS for the DK-DNR, since there were not enough time points available after the 2018 RMMs for this data source, but their incidence pre-intervention was 0.2/1000 PM, and post-intervention 0.26/1000 PM. Upon stratification by indication, isotretinoin had the highest incidence of use, and alitretinoin the lowest, but without a significant level or trend change following implementation of RMMs in 2018. Only in IT-ARS, a significant downward trend change of −0.0002 new users/1000 PM (*p* = 0.04) was seen for incident use of acitretin for psoriasis.

No statistically significant post-intervention level and trend changes were detected for discontinuation rates in the ITS analyses, see Supplementary Figure S2. Reasons for discontinuation were mostly unknown (>95%) across all databases. Due to seasonality, most data sources presented a higher discontinuation rate during summer months (higher than 15% per month in ES-VID, IT-ARS and ES-BIFAP and higher than 30% in IT-Caserta). No relevant differences were found in the sensitivity analysis when redefining the discontinuation gaps.

### Contraception use while retinoid exposure and 90 days prior retinoid start

**FIGURE 3:**
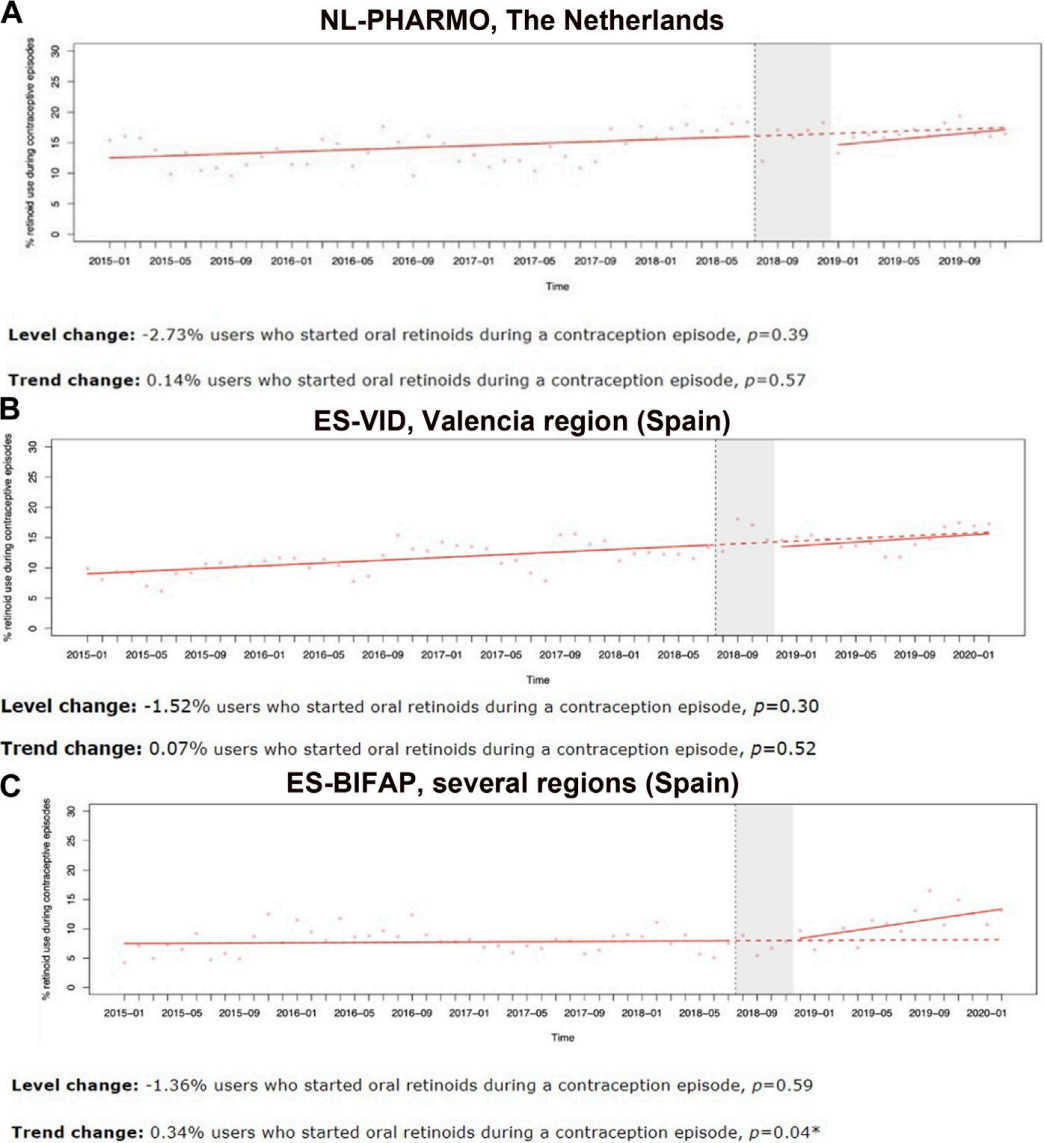
Interrupted time-series analyses (ITSA) on the monthly percentage of oral retinoid users who start within a contraception coverage episode, in female subjects of childbearing age per database, between 2010 and 2020, excluding COVID-19 pandemic period. **(A)** NL-PHARMO, Netherlands. **(B)** ES-VID, Valencia region (Spain). **(C)** ES-BIFAP, several regions (Spain).

Identification of contraceptive use was limited, especially in DK-DNR, IT-ARS and IT-Caserta, where ITS analyses could not be performed. In NL-PHARMO and ES-VID, the percentage of retinoid prescription/dispensing happening during a contraception episode showed a gradual increase over time, going from 5% to 29%, and from 1% to 24%, respectively. However, the ITS analyses showed no significant level or trend change after implementation of 2018 RMMs (Figure 3). In ES-BIFAP, not only percentages increased but also the ITS analyses showed a significant trend increase after the 2018 RMMs in both the percentage of retinoids initiated within a recorded contraception episode (trend change =0.34%, *p* = 0.04) (Figure 3) and contraceptives initiated 90 days prior to the start of a retinoid (Supplementary Figure S3).

### Pregnancy testing prior and during retinoid exposure

DK-DNR, NL-PHARMO, IT-ARS and IT-Caserta were not able to capture pregnancy tests. In ES-BIFAP hardly any pregnancy test was identified. In ES-VID, the average monthly rate of recorded pregnancy test prior to a retinoid dispensing/prescription was 5.3% between 2015 and the implementation of the revised RMMs in 2018 and 4.7% after the policy. Therefore, we could not model ITS analyses on pregnancy testing data.

### Pregnancy occurrence overall and relating to retinoid exposure

Concomitant pregnancies together with retinoid use were identified in all databases. Table 2 shows i) the rates of retinoids prescribed during a pregnancy, and ii) the rates of pregnancy occurrence while being treated with an oral retinoid, both pre and post 2018 RMMs. Post-intervention rates of retinoid use during pregnancy decreased across all DAPs and the rate difference was significant in ES-VID and IT-Caserta. Second, in pregnancies identified during treatment with an oral retinoid, the post-intervention rates decreased across all DAPs (significant in ES-VID), except in ES-BIFAP, where the rate increased but was non-significant. In the sensitivity analysis run in IT-ARS (red pregnancies), pre- and post-intervention pregnancy rates were higher than in the main analysis (Supplementary Table S3).

**TABLE 2:** Pregnancy counts and rates for pregnancies occurring during a retinoid exposure, and retinoid exposures starting during pregnancy.

i) Retinoid use during pregnancy							
	Pre 2018 revision RMM			Post 2018 revision RMM			
	Cases	Users^b^	Rate^a^	Cases	Users^b^	Rate^a^	RD post-pre (CI 99%)
NL-PHARMO	18	47,521	0.38	<5	12,893	0.16	−0.22 (−0.5 to 0.1)
IT-ARS	7	38,189	0.18	<5	15,823	0.13	−0.05 (−0.3 to 0.2)
IT-Caserta	7	27,307	0.22	0	11,677	0	−0.22 (−0.4 to −0.1)
ES-VID	88	117,247	0.75	15	36,055	0.42	−0.33 (−0.6 to −0.1)
ES-BIFAP	10	57,217	0.17	<5	18,244	0.11	−0.06 (−0.3 to 0.1)

ii) Pregnancy during retinoid use							
	Pre 2018 revision RMM			Post 2018 revision RMM			
	Cases	Users^b^	Rate^a^	Cases	Users^b^	Rate^a^	RD post-pre (CI 99%)
NL-PHARMO	38	47,521	0.80	<5	12,893	0.23	−0.57 (-0.9 to −0.2)
IT-ARS	15	38,189	0.39	<5	15,823	0.25	−0.14 (−0.5 to 0.2)
IT-Caserta	6	27,307	0.60	0	11,677	0	−0.22 (−0.4 to 0)
ES-VID	82	117,247	0.69	14	36,055	0.39	−0.31 (−0.6 to −0.1)
ES-BIFAP	7	57,217	0.12	<5	18,244	0.22	+0.10 (−0.1–0.3)

RD, rate difference.

^a^ Rates per 1,000 users, numerators include only pregnancies classified as green (recorded end and start date, or yellow: start data imputed from recorded end date).

^b^ Prevalent users of oral retinoids.

## Discussion

The aim of this study was to measure the impact of the revised and implemented 2018 RMMs on utilisation patterns of oral retinoids, contraceptive measures, pregnancy testing, and occurrence of pregnancy during retinoid treatment in four European countries. Our key findings show that utilisation of retinoids did not change after the implementation of 2018 RMM, and that pregnancies keep occurring concomitantly to retinoids use, both before and after the 2018 RMMs. Contraceptive use and pregnancy testing could not be reliably captured and therefore, not assessed in most of the databases. In Spain (BIFAP-ES), concurrent use of contraceptives and oral retinoids increased after the implementation of the RMMs in 2018.

The main goal of the PPP is to avoid pregnancies during an oral retinoid treatment, including risk periods before and after treatment. After the 2003 PPP was released, its effectiveness was reviewed and despite a reduction in numbers, pregnancies exposed to retinoids have continued to occur (Teichert et al., 2010; Crijns et al., 2012; Zomerdijk et al., 2014; Raguideau et al., 2015; Henry et al., 2016). Furthermore, we showed that pregnancies temporarily related to retinoid use still occurred even after implementation of the 2018 RMMs, and most importantly, retinoid treatment events starting during pregnancy were captured. In fact, a systematic review to check compliance to the 2003 PPP in Europe (Crijns et al., 2011) showed that only 6%–26% isotretinoin was prescribed in full accordance with the PPP. Pregnancy incidence was seen in 0.2–1.0 per 1,000 females of childbearing age using isotretinoin, and these figures align with our post-2018 RMMs rates. Therefore, the PPPs in Europe, as in the rest of the world seem to be insufficient, despite successive modifications (Kovitwanichkanont and Driscoll, 2018; Choi and Han, 2021; Cha et al., 2022).

In general, our results show a slight increase in the utilisation patterns of oral retinoids over the study period, with non-significant changes after the implementation of the RMM in 2018. Although only few studies have been conducted to report utilisation figures in the general female population, those studies have found a similar upward utilisation trend of oral retinoids within the past 3 decades (Khiali et al., 2018). The fact of not finding a significant utilisation decrease, both immediately and over time after the implementation of the 2018 RMMs, could denote the difficulty of aligning and implementing European RMMs into prescribers’ daily clinical practice, as reported elsewhere (Leonardo Alves et al., 2022). Furthermore, monthly incidence and prevalence rates showed that retinoid prescriptions have a strong seasonal pattern, with decreasing incidence in the summer months, especially in southern countries. This seasonal pattern is aligned with clinical practice recommendations to carefully use retinoids with sunlight due to photosensitivity (Ferguson and Johnson, 1989), and potentially different disease severity across seasons.

Only few or no records of pregnancy tests were detected before and after the initiation of an oral retinoid. Besides this recording issue, survey-based studies performed in some European countries have reported only 15% of requests of negative pregnancy tests among female retinoid users by healthcare professionals (Crijns et al., 2013; Lelubre et al., 2018). Contraception use at start of or during a retinoid treatment was recorded very differently across databases, since several non-user/non-permanent contraceptive measures are not recorded because of lack of reimbursement or over-the-counter (OTC) status. We could investigate trends of contraceptive coverage in Netherlands and Spain, and only in ES-BIFAP a significant increase in use of contraceptive use was shown after the 2018 RMM. One explanatory factor for pregnancy occurrence during a retinoid treatment is the lack of contraceptive use in 70% of patients (Schaefer et al., 2010). Although there is a strong awareness regarding the teratogenic risks of oral retinoids among patients, prescribers, and pharmacists in eight European countries (Leonardo Alves et al., 2022), there was moderate awareness about the RMMs and educational materials. Furthermore, the lack of registration of contraception use and pregnancy testing in EHDs jeopardises a proper policy impact assessment.

### Strengths and limitations

This study has several strengths. First, the total sample size of more than 10.5 million female subjects of child-bearing potential permitted the calculation of precise estimates of oral retinoid utilisation and other outcomes on a monthly basis. In addition, analysing the implementation of the 2018 RMMs across four different countries allowed to examine the potential impacts between countries based on the differences in how and when the 2018 RMMs were implemented. Moreover, using several databases from different countries across Europe has led to a large and diverse study population with good representativeness, and generalisable results to a European setting. Second, a rigorous quality checking process enabled this study to detect data uncertainties or issues in the analyses. Third, use of the ConcePTION CDM based on a common protocol was key to minimising unwanted heterogeneity in the results due to differences in the implementation of the study. Fourth, we applied ITS analyses to evaluate the impact of the 2018 RMMs on the outcomes of interest. This is the strongest quasi-experimental design and the gold standard method for policy analysis in drug utilisation research (Grimshaw et al., 2000; Wagner et al., 2002; Jandoc et al., 2015).

Regarding the limitations of our study, ES-BIFAP is restricted to mainly prescription/dispensing information from primary care, this may result in incomplete information on retinoids use. In general, misclassification of exposure or outcome may occur, affecting the recording of contraceptive measures (especially oral contraceptives and barrier methods), the detection of folic acid as proxy of pregnancy wish, and the identification of pregnancy tests. To mitigate this, we implemented a pregnancy detection algorithm. In the main analysis, pregnancies whose end date, or end and start date were used and validated. As a birth is more likely to produce a more specific recording of the date of end of the pregnancy event, pregnancies with an uncertain date of end such as spontaneous abortions, or elective terminations could be prone to be missed. In this sense, we conducted a targeted sensitivity analysis in IT-ARS to include red pregnancies (imputation of start and end date). It showed a potential underestimation of the pregnancy rates from missing pregnancies belonging to that categories (early termination). In fact, pregnancy outcomes should be studied in future research. Moreover, as we required an end date of pregnancy in the main analysis, we may have misclassification to the end of follow-up, since pregnancies that may have started post-RMMs may not have ended, and therefore could not be identified.

The occurrence of the COVID-19 pandemic was another obstacle in conducting this research. Almost all countries went into periods of partial or full lockdown in 2020, which certainly affected not only the healthcare resources, but also access to health services, and patients’ behaviour, especially their attitude towards repeated prescriptions. To overcome this issue, we excluded the COVID-19 period from our main analyses. Finally, unmeasured time-varying confounders may exist, especially when we did not have any control group or comparison outcome (Grimshaw et al., 2000).

## Conclusion

This study shows a limited impact of the 2018 RMMs on oral retinoids utilisation patterns and pregnancy prevention measures among females of child-bearing age in four European countries. Like previous studies assessing former RMMs in Europe, we observed that pregnancies still occur during retinoid use, and oral retinoids are still prescribed to pregnant women. Our results challenge the effectiveness of PPPs. Regulators, policymakers, prescribers, and researchers must rethink implementation strategies to avoid any pregnancy temporarily related to a retinoid prescription. The target of a retinoid’s PPP must be zero exposed pregnancies.

## Data Availability

The datasets presented in this article are not readily available because this study used anonymised patient data from electronic health care databases in different countries/regions. These data remained local within each centre as local data protection regulations for individual data privacy protection apply. Requests to access the datasets should be directed to the corresponding author CD at c.e.duransalinas@umcutrecht.nl.

## Supplementary material

SUPPLEMENTARY TABLE S1 Overview of databases used in this study

SUPPLEMENTARY TABLE S2 Retinoid associated adverse drug reactions (ADRs)

SUPPLEMENTARY TABLE S3 Pregnancy counts and rates for pregnancies occurring during and retinoid exposure, and retinoid exposures starting during pregnancy, in ARS: main analysis compared with sensitivity analysis, and impact of adding ‘red’ pregnancies to each stratum of pregnancies.

SUPPLEMENTARY FIGURE S1 Query diagram to illustrate treatment episode construction and treatment discontinuation definition of oral retinoid and contraceptive exposures.

SUPPLEMENTARY FIGURE S2 Interrupted time-series analyses (ITSA) on monthly percentage of oral retinoid discontinuers per database, between 2010 and 2020, excluding COVID-19 pandemic period.

SUPPLEMENTARY FIGURE S3 Interrupted time-series analyses (ITSA) on monthly proportion of retinoid users who started a contraceptive treatment within 90 days before to start an oral retinoid therapy over all retinoid users that month. Numerator is the number of persons who were prescribed or dispensed a contraceptive within 90 days prior the actual date of start of the retinoid therapy, in female subjects of childbearing age per database, between 2010 and 2020, excluding COVID-19 pandemic period.

## References

[B1] Cha E. H. Kim N. Kwak H. S. Han H. J. Joo S. H. Choi J. S. (2022). Pregnancy and neonatal outcomes after periconceptional exposure to isotretinoin in Koreans. Obstet. Gynecol. Sci. 65 (2), 166–175. 10.5468/ogs.21354 35193174 PMC8942757

[B2] Choi E. J. Han J. Y. (2021). Non-compliance with pregnancy prevention recommendations for isotretinoin in Korea between 2019-2020. Obstet. Gynecol. Sci. 64 (2), 201–208. 10.5468/ogs.20247 33752279 PMC7990996

[B3] Crijns H. J. Straus S. M. Gispen-de Wied C. de Jong-van den Berg L. T. W. (2011). Compliance with pregnancy prevention programmes of isotretinoin in Europe: A systematic review. Br. J. Dermatol 164 (2), 238–244. 10.1111/j.1365-2133.2010.09976.x 20716214

[B4] Crijns H. J. van Rein N. Gispen-de Wied C. C. Straus S. M. de Jong-van den Berg L. T. (2012). Prescriptive contraceptive use among isotretinoin users in The Netherlands in comparison with non-users: A drug utilisation study. Pharmacoepidemiol Drug Saf. 21, 1060–1066. 10.1002/pds.3200 22228673

[B5] Crijns I. Mantel-Teeuwisse A. Bloemberg R. Pinas E. Straus S. de Jong-van den Berg L. (2013). Healthcare professional surveys to investigate the implementation of the isotretinoin pregnancy prevention programme: A descriptive study. Expert Opin. Drug Saf. 12 (1), 29–38. 10.1517/14740338.2013.745850 23163396

[B6] Dai W. S. LaBraico J. M. Stern R. S. (1992). Epidemiology of isotretinoin exposure during pregnancy. J. Am. Acad. Dermatol 26 (4), 599–606. 10.1016/0190-9622(92)70088-W 1597546

[B7] Durbin J. Watson G. S. (1950). Testing for serial correlation in least squares regression. I. I. Biom. 37 (3-4), 409–428. 10.1093/biomet/37.3-4.409 14801065

[B8] European Medicines Agency (2018), Updated measures for pregnancy prevention during retinoid use. Available at: https://www.ema.europa.eu/en/news/updated-measures-pregnancy-prevention-during-retinoid-use (Accessed 23 March 2018)

[B9] Ferguson J. Johnson B. E. (1989). Retinoid associated phototoxicity and photosensitivity. Pharmacol. Ther. 40 (1), 123–135. 10.1016/0163-7258(89)90079-x 2645585

[B10] García-Sempere A. Orrico-Sánchez A. Muñoz-Quiles C. Hurtado I. Peiró S. Sanfélix-Gimeno G. (2020). Data resource profile: the Valencia health system integrated database (VID). Int. J. Epidemiol. 49 (3), 740–741e. 10.1093/ije/dyz266 31977043 PMC7394961

[B11] Gini R. Bartolini C. Limoncella G. Paoletti O. Messina D. Cid A. (2022). ConcePTION algorithm pregnancies. Available at: https://github.com/ARS-toscana/ConcePTIONAlgorithmPregnancies.git .

[B12] Grimshaw J. Campbell M. Eccles M. Steen N. (2000). Experimental and quasi-experimental designs for evaluating guideline implementation strategies. Fam. Pract. 17 (Suppl. 1), S11–S16. 10.1093/fampra/17.suppl_1.s11 10735262

[B13] Henry D. Dormuth C. Winquist B. Carney G. Bugden S. Teare G. (2016). Occurrence of pregnancy and pregnancy outcomes during isotretinoin therapy. CMAJ 188, 723–730. 10.1503/cmaj.151243 27114489 PMC4938682

[B14] Jandoc R. Burden A. M. Mamdani M. Lévesque L. E. Cadarette S. M. (2015). Interrupted time series analysis in drug utilization research is increasing: systematic review and recommendations. J. Clin. Epidemiol. 68 (8), 950–956. 10.1016/j.jclinepi.2014.12.018 25890805

[B15] Khiali S. Gharekhani A. Entezari-Maleki T. (2018). Isotretinoin; A review on the utilization pattern in pregnancy. Adv. Pharm. Bull. 8 (3), 377–382. 10.15171/apb.2018.044 30276133 PMC6156490

[B16] Kovitwanichkanont T. Driscoll T. (2018). A comparative review of the isotretinoin pregnancy risk management programs across four continents. Int. J. Dermatol 57, 1035–1046. 10.1111/ijd.13950 29508918

[B17] Kuiper J. G. Bakker M. Penning-Van Beest F. J. A. Herings R. M. C. (2020). Existing data sources for clinical epidemiology: the PHARMO database network. Clin. Epidemiol. 12, 415–422. 10.2147/CLEP.S247575 32425609 PMC7196787

[B18] Lelubre M. Hamdani J. Senterre C. Amighi K. Peres M. Schneider M. P. (2018). Evaluation of compliance with isotretinoin PPP recommendations and exploration of reasons for non-compliance: survey among French-speaking health care professionals and patients in Belgium. Pharmacoepidemiol Drug Saf. 27 (6), 668–673. 10.1002/pds.4441 29726056

[B19] Leonardo Alves T. Hegger I. Almasdottir A. B. Heerdink R. Bouvy M. Lahousse L. (2022). Measuring awareness and use of pregnancy prevention measures for valproate and retinoid containing products among patients, prescribers and pharmacists across eight European countries.

[B20] Maciá-Martínez M. A. Gil M. Huerta C. Martín-Merino E. Álvarez A. Bryant V. (2020). Base de Datos para la Investigación Farmacoepidemiológica en Atención Primaria (BIFAP): A data resource for pharmacoepidemiology in Spain. Pharmacoepidemiol Drug Saf. 29 (10), 1236–1245. 10.1002/pds.5006 32337840

[B21] Matcho A. Ryan P. Fife D. Gifkins D. Knoll C. Friedman A. (2018). Inferring pregnancy episodes and outcomes within a network of observational databases. PLoS One 13 (2), e0192033. 10.1371/journal.pone.0192033 29389968 PMC5794136

[B22] Panchaud A. Csajka C. Merlob P. Schaefer C. Berlin M. De Santis M. (2012). Pregnancy outcome following exposure to topical retinoids: A multicenter prospective study. J. Clin. Pharmacol. 52, 1844–1851. 10.1177/0091270011429566 22174426

[B23] Pinheiro S. P. Kang E. M. Kim C. Y. Governale L. A. Zhou E. H. Hammad T. A. (2013). Concomitant use of isotretinoin and contraceptives before and after iPledge in the United States. Pharmacoepidemiol Drug Saf. 22 (12), 1251–1257. 10.1002/pds.3481 23913625

[B24] Raguideau F. Mezzarobba M. Zureik M. Weill A. Ricordeau P. Alla F. (2015). Compliance with pregnancy prevention plan recommendations in 8672 French women of childbearing potential exposed to acitretin. Pharmacoepidemiol Drug Saf. 24, 526–533. 10.1002/pds.3763 25753265

[B25] Retinoids (2018). Retinoids: EMA updated warnings. Drug Ther. Bull. 56, 40. 10.1136/dtb.2018.4.0608 29618570

[B26] Schaefer C. Meister R. Weber-Schoendorfer C. (2010). Isotretinoin exposure and pregnancy outcome: an observational study of the berlin institute for clinical teratology and drug risk assessment in pregnancy. Arch. Gynecol. Obstet. 281, 221–227. 10.1007/s00404-009-1112-2 19444462

[B27] Shin J. Cheetham T. C. Wong L. Niu F. Kass E. Yoshinaga M. A. (2011). The impact of the iPLEDGE program on isotretinoin fetal exposure in an integrated health care system. J. Am. Acad. Dermatol 65 (6), 1117–1125. 10.1016/j.jaad.2010.09.017 21565419

[B28] Soprano D. R. Soprano K. J. (1995). Retinoids as teratogens. Annu. Rev. Nutr. 15, 111–132. 10.1146/annurev.nu.15.070195.000551 8527214

[B29] Teichert M. Visser L. E. Dufour M. Rodenburg E. Straus S. M. De Smet P. A. (2010). Isotretinoin use and compliance with the Dutch pregnancy prevention programme: A retrospective cohort study in females of reproductive age using pharmacy dispensing data. Drug Saf. 33, 315–326. 10.2165/11319190-000000000-00000 20297863

[B30] Thurin N. H. Pajouheshnia R. Roberto G. Dodd C. Hyeraci G. Bartolini C. (2022). From inception to ConcePTION: genesis of a network to support better monitoring and communication of medication safety during pregnancy and breastfeeding. Clin. Pharmacol. Ther. 111 (1), 321–331. 10.1002/cpt.2476 34826340 PMC9299060

[B31] Wagner K. Soumerai S. B. Zhang F. Ross-Degnan D. (2002). Segmented regression analysis of interrupted time series studies in medication use research. J. Clin. Pharm. Ther. 27, 299–309. 10.1046/j.1365-2710.2002.00430.x 12174032

[B32] Zomerdijk I. M. Ruiter R. Houweling L. M. Herings R. M. Sturkenboom M. C. Straus S. M. (2014). Isotretinoin exposure during pregnancy: A population-based study in The Netherlands. BMJ Open 4, e005602. 10.1136/bmjopen-2014-005602 PMC4244495 25392022

