# Supplementary figures and images for: Impact of the 2018 revised Pregnancy Prevention Programme by the European Medicines Agency on the use of oral retinoids in females of childbearing age in Denmark, Italy, Netherlands, and Spain: an interrupted time series analysis
Source: Front Pharmacol. 2023 Aug 17;14:1207976. doi: 10.3389/fphar.2023.1207976 (PMC10469888; doi:10.3389/fphar.2023.1207976)

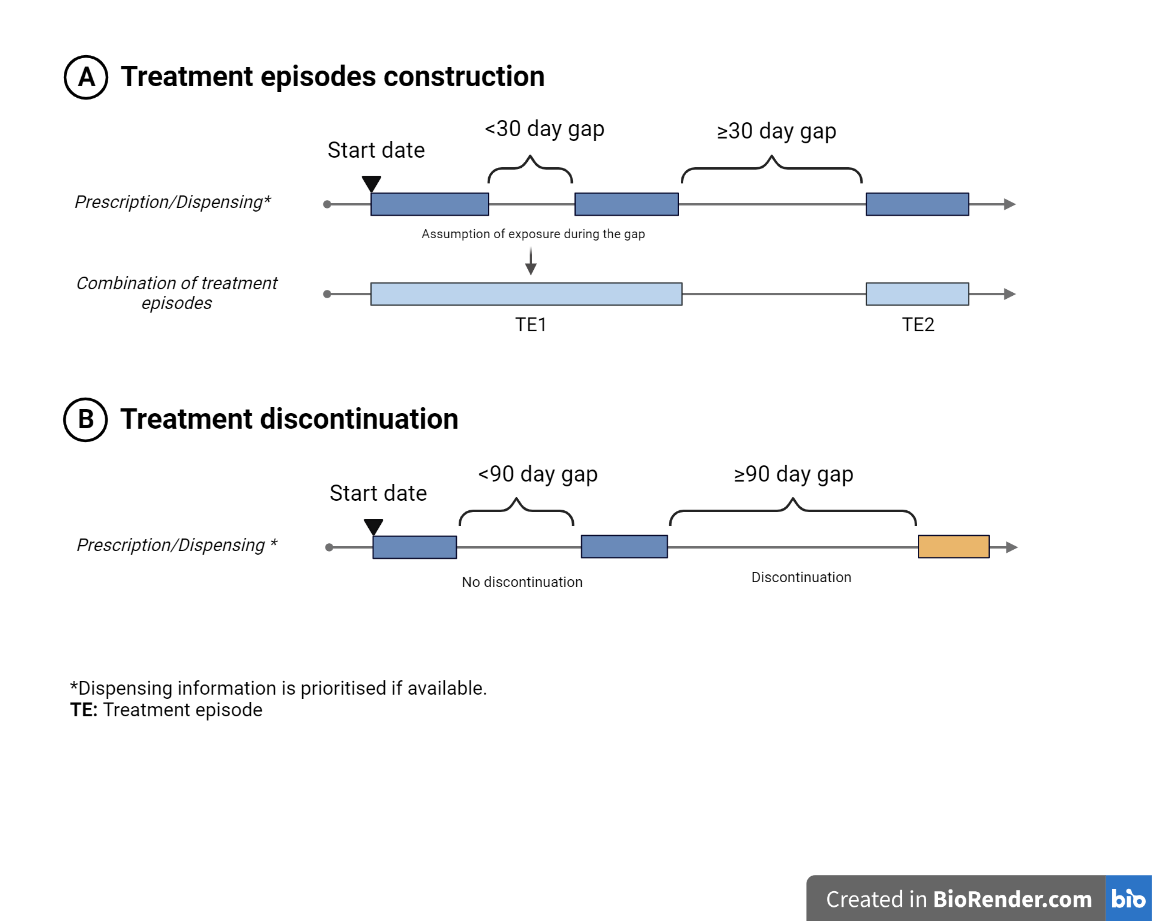

Supplement: Supplementary file 2 [file Image1.tiff]
