# Supplementary material for: Impact of the 2018 revised Pregnancy Prevention Programme by the European Medicines Agency on the use of oral retinoids in females of childbearing age in Denmark, Italy, Netherlands, and Spain: an interrupted time series analysis
Source: Front Pharmacol. 2023 Aug 17;14:1207976. doi: 10.3389/fphar.2023.1207976 (PMC10469888; doi:10.3389/fphar.2023.1207976)
Supplement: Supplementary file 3 [file Table1.docx]

**SUPPLEMENTARY TABLE S1: Overview of databases used in this study**

| Characteristic | NL-PHARMO  Nationally representative | DK-Danish National Registries* | IT-ARS  Tuscany | ES-BIFAP  Multi-regional† | ES-VID  Valencia | IT-Caserta  Campania |
| --- | --- | --- | --- | --- | --- | --- |
| Data access provider | PHARMO | University of Copenhagen | ARS | AEMPS | FICF/FISABIO | University Messina |
| Country  (pop. size in million) | Netherlands (17.0) | Denmark  (5.8) | Italy  (59.8) | Spain  (46.5) | Spain  (46.5) | Italy  (59.8) |
| Type of database | EMR | ADM | ADM | EMR | EMR | ADM |
| # in DB | 4.2 million (prior to linkage) | 5.8 million | 3.6 million | 9 million | 5.1 million | 0.9 million |
| GP Rx | Yes | Yes | Yes | Yes | Yes | Yes |
| Outpatient Rx spec. | Yes | Yes | Yes | No | Yes | Yes |
| Private Rx | No | No | No | No | No | No |
| Inpatient hospital Rx | Yes (not utlilised in this study) | No | No | No | No | No |
| Date of Rx | Yes | No | No | Yes | Yes | Yes |
| Date of Dispensing | Yes | Yes | Yes | Yes | Yes |  |
| Quantity | Yes | Yes | Yes | Yes | Yes | Yes |
| Duration | Yes | Yes | DDD based | Yes | Yes | DDD based |
| Strength | Yes | Yes | Yes | Yes | Yes | Yes |
| Coding of drugs | ATC | ATC | ATC | ATC | ATC | ATC |
| Oral contraceptives | Yes | Yes | No | Yes (only those dispensed with a prescription) | Yes (only those publicly funded) | No |
| Observed pregnancy test dx | No | No | No | No | Unclear for retinoids | Unclear for retinoids |
| Date fitted IUD | Yes | Date of prescription fill (not copper IUD) | No | No | No | No |
| Date removed IUD | Yes | No | No | No | No | No |
| Hysterectomy | Yes | Yes | Yes | If recorded by GP | Yes | Yes |
| Oophorectomy | Yes | Yes | Yes | If recorded by GP | Yes | Yes |
| Sterilization | Yes | Yes | Yes | If recorded by GP | Yes | Yes |
| Partner vasectomy | No | No | No | No | No | No |
| Coding of disease | ICPC, ICD-9, ICD-10 | ICD-10 | ICD-9 CM/ICD-10 | ICD-9, SNOMED, ICD-10 | ICD-9CM/ICD-10CM | ICD-9CM |
| Pregnancy outcomes | Linkage to birth register | Linkage to birth register | Linkage to birth register /interruption registry/spontaneous abortions registry | Mother’s records if recorded by the GP and in hospital discharge record | Linkage to perinatal registry | Mother’s records |

ADM = Administrative; ATC = Anatomical Therapeutic Chemical; DDD=defined daily dose; EMR = Electronic Medical Records; ICD= International Classification of Disease; ICPC = International Classification of Primary Care; Rx = prescriptions/dispensations.

* Due to restrictions caused by COVID-19 pandemic, a limited dataset was used as the Danish data source for this study. This limited dataset was from an existing project on evaluation of risk minimisation measures and included only data from the Danish National Prescription Registry. This caused several limitations to various sections of this study including data acquisition and analysis, as the following:

- *Study population:* All females of childbearing age with retinoid dispensing (aged 12-55 years) resident in Denmark between 01.01.2010 and 31.12.2018 were included, as data from the originally intended last 2 years of study period (i.e., 2019 and 2020) were not available. Due to this limitation, no ITS analyses could be performed due to the few time points available after the implementation of 2018 risk minimisation measures in Denmark. The Danish female population of childbearing age (including non-retinoid users) and thus all the related denominators in various analyses of this study (such as for calculating incidence or prevalence rates) was estimated based on public Danish population numbers from Statistics Denmark.
- *Retinoids available:* acitretin was not available in the data used for this study.
- *Contraception:* User-independent permanent methods of contraception were not available in the data used for this study.
- *Pregnancy wish: folic acid use was not available in the data used for this study.*
- *Pregnancy:* information about pregnancies and pregnancy testing was not captured in the data used for this study.

†Nine out of eleven regions were included in BIFAP.
