# Supplementary material for: Impact of the 2018 revised Pregnancy Prevention Programme by the European Medicines Agency on the use of oral retinoids in females of childbearing age in Denmark, Italy, Netherlands, and Spain: an interrupted time series analysis
Source: Front Pharmacol. 2023 Aug 17;14:1207976. doi: 10.3389/fphar.2023.1207976 (PMC10469888; doi:10.3389/fphar.2023.1207976)
Supplement: Supplementary file 4 [file Image2.pdf]

Figure S2. Interrupted time-series analyses (ITSA)* on monthly percentage of oral retinoid discontinuers per database, between 2010 and 2020, excluding COVID-19 pandemic period.

Figure 2a. NL-PHARMO, The Netherlands

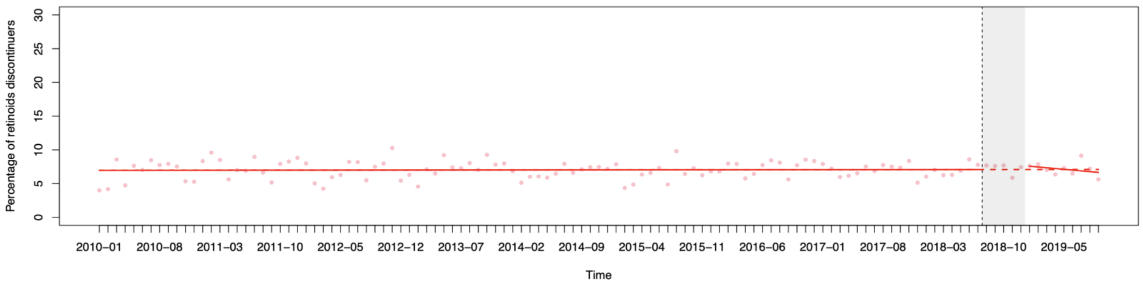

Level change: 1.18% retinoids discontinuers, *p*=0.50

Trend change: -0.11% retinoids discontinuers, *p*=0.50

Figure 2b. IT-ARS, Tuscany (Italy)

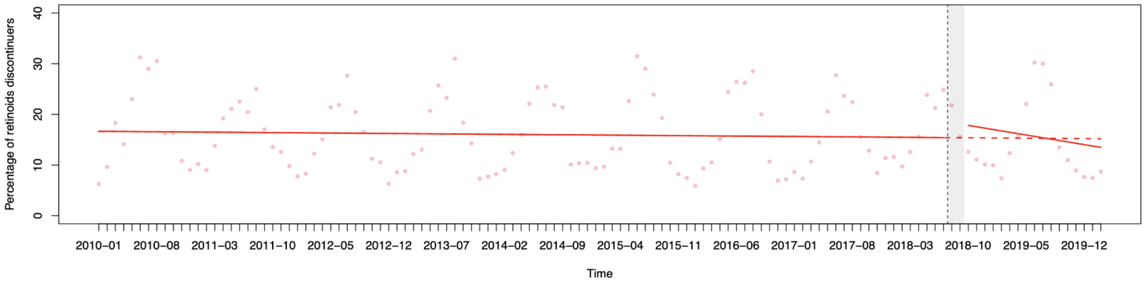

Level change: 2.69% retinoids discontinuers, *p*=0.30

Trend change: -0.25% retinoids discontinuers, *p*=0.27

Figure 2c. IT-Caserta region (Italy) [T]

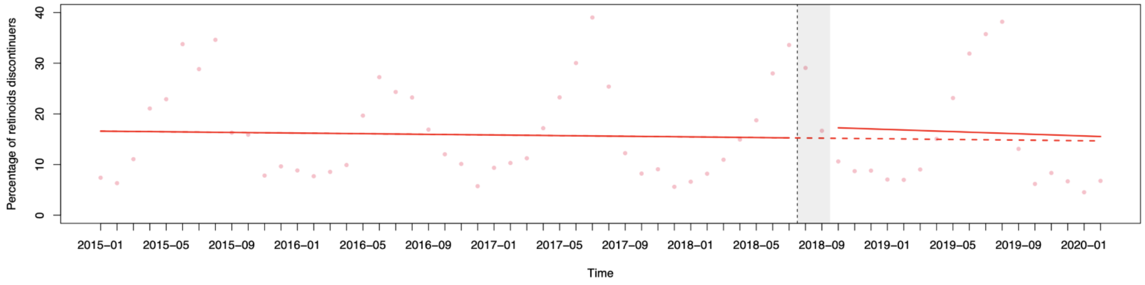

Level change: 2.15% retinoids discontinuers, *p*=0.35

Trend change: -0.07% retinoids discontinuers, *p*=0.67

Figure 2d. ES-VID, Valencia region (Spain)

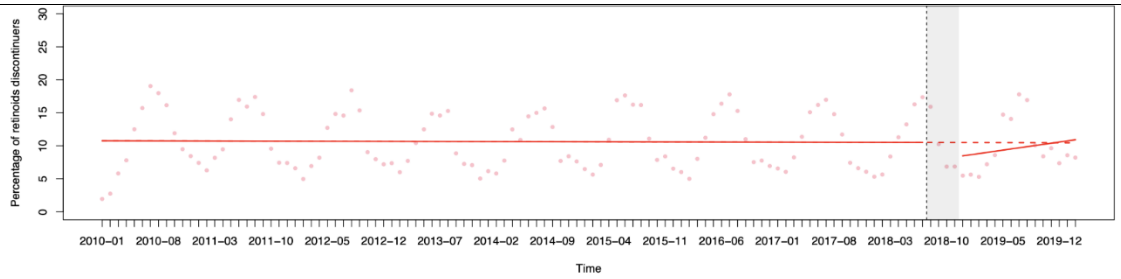

Level change: -2.19% retinoids discontinuers, *p*=0.380

Trend change: 0.17% retinoids discontinuers, *p*=0.385

Figure 2e. ES-BIFAP, several regions (Spain)

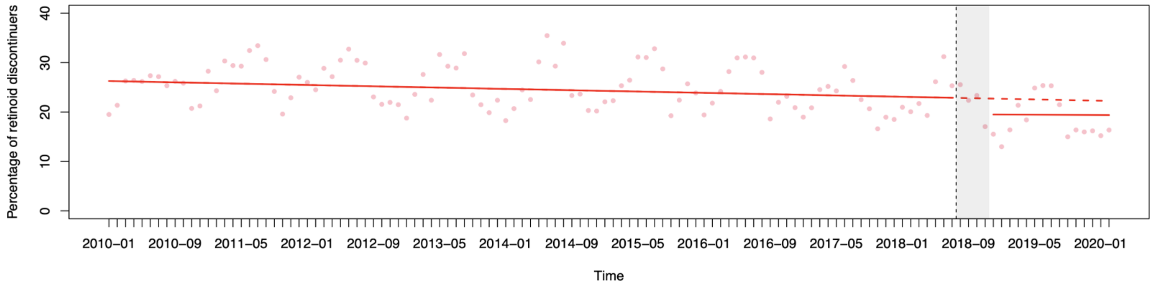

Level change: -3.24% retinoid discontinuers, *p*=0.07

Trend change: 0.025% retinoids discontinuers, *p*=0.85

*Level change shows the impact of the RMM implementation by measuring the distance between the projection of the slope from the period prior to implementation and the starting point of the slope directly after the implementation. Trend change measures the difference between the points of the observed real post-intervention estimates and the predicted estimates based on projection of the slope prior to the intervention.

[T] For Caserta ITS, analysis was restricted to 2015-2020 because of change in data capturing.
