# Supplementary material for: Impact of the 2018 revised Pregnancy Prevention Programme by the European Medicines Agency on the use of oral retinoids in females of childbearing age in Denmark, Italy, Netherlands, and Spain: an interrupted time series analysis
Source: Front Pharmacol. 2023 Aug 17;14:1207976. doi: 10.3389/fphar.2023.1207976 (PMC10469888; doi:10.3389/fphar.2023.1207976)
Supplement: Supplementary file 5 [file Image3.pdf]

Figure S3. Interrupted time-series analyses (ITSA)* on monthly proportion of retinoid users who started a contraceptive treatment within 90 days before to start an oral retinoid therapy over all retinoid users that month. Numerator is the number of persons who were prescribed or dispensed a contraceptive within 90 days prior the actual date of start of the retinoid therapy, in female subjects of childbearing age per database, between 2010 and 2020, excluding COVID-19 pandemic period[T &]

Figure 3a. NL-PHARMO, The Netherlands

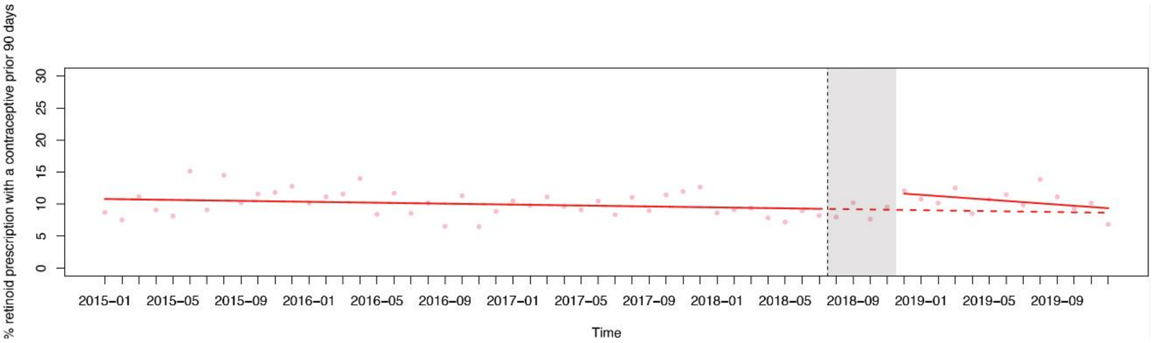

Level change: 3,30%, p=0.07

Trend change: -0.15%, p=0.30

Figure 3b. ES-VID, Valencia region (Spain)

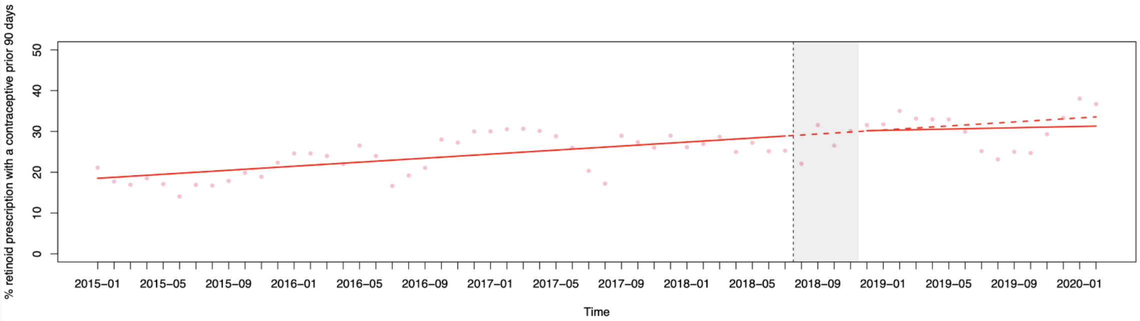

Level change: 0,90%, p=0.83

Trend change: -0.16%, p=0.59

Figure 3c. ES-BIFAP, several regions (Spain)

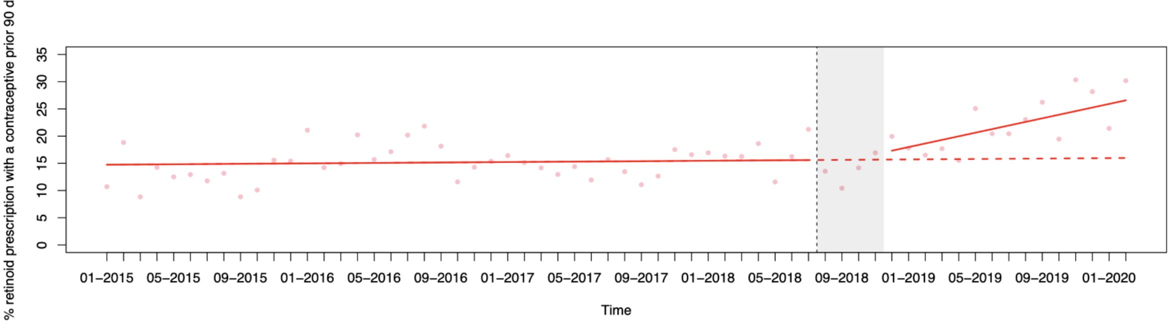

Level change: -1.5%, p=0.59

Trend change: 0.64%, p=0.004*

*Level change shows the impact of the RMM implementation by measuring the distance between the projection of the slope from the period prior to implementation and the starting point of the slope directly after the implementation. Trend change measures the difference between the points of the observed real post-intervention estimates and the predicted estimates based on projection of the slope prior to the intervention.

[T] For IT-ARS and DK-DNR , ITS could not be performed because contraceptive use could not be assessed.

[&] For IT-Caserta ITS, the percentages of recorded contraceptive prescriptions within 90 days prior to a retinoid dispensing was very low, so ITS could not be performed.
