# Supplementary material for: Impact of the 2018 revised Pregnancy Prevention Programme by the European Medicines Agency on the use of oral retinoids in females of childbearing age in Denmark, Italy, Netherlands, and Spain: an interrupted time series analysis
Source: Front Pharmacol. 2023 Aug 17;14:1207976. doi: 10.3389/fphar.2023.1207976 (PMC10469888; doi:10.3389/fphar.2023.1207976)
Supplement: Supplementary file 6 [file Table3.docx]

**SUPPLEMENTARY TABLE S3.** Pregnancy counts and rates for pregnancies occurring during and retinoid exposure, and retinoid exposures starting during pregnancy, in ARS: main analysis compared with sensitivity analysis, and impact of adding ‘red’ pregnancies to each stratum of pregnancies

|  | i) Retinoid use during pregnancy | | | | | | |
| --- | --- | --- | --- | --- | --- | --- | --- |
|  | Pre 2018 revision RMM | | | Post 2018 revision RMM | | | |
|  | Cases | Users^†^ | Rate* | Cases | Users^†^ | Rate * | RD post-pre (CI 99%) |
| IT-ARS | 7 | 38189 | 0.18 | <5 | 15823 | 0.13 | -0.05 (-0.3 to 0.2) |
| IT-ARS – sensitivity | 20 | 38189 | 0.52 | <5 | 15823 | 0.19 | -0.75 (-0.75 to 0.08) |
| Impact of added pregnacies^&^ |  |  | **+189%** |  |  | **+50%** |  |

|  | ii) Pregnancy during retinoid use | | | | | | |
| --- | --- | --- | --- | --- | --- | --- | --- |
|  | Pre 2018 revision RMM | | | Post 2018 revision RMM | | | |
|  | Cases | Users^†^ | Rate* | Cases | Users^†^ | Rate * | RD post-pre (CI 99%) |
| IT-ARS | 15 | 38189 | 0.39 | <5 | 15823 | 0.25 | -0.14 (-0.5 to 0.2) |
| IT-ARS – sensitivity | 19 | 38189 | 0.50 | 6 | 15823 | 0.38 | -0.61 (-0.61;0.38) |
| Impact of added pregnacies ^&^ |  |  | **+46%** |  |  | **+52%** |  |

*RD: Rate difference*

**Rates per 1000 users, numerators include only pregnancies classified as green (recorded end and start date, or yellow: start data imputed from recorded end date)*

^†^ *Prevalent users of oral retinoids*

*^&^Impact calculated as 100 x ((rate sensitivity/rate main) – 1)*
